# Supplementary material for: Molecular pathways of senescence regulate placental structure and function
Source: EMBO J. 2019 Aug 19;38(18):e100849. doi: 10.15252/embj.2018100849 (PMC6745498; doi:10.15252/embj.2018100849)
Supplement: Supplementary file 1 — Appendix [file EMBJ-38-e100849-s001.pdf]

APPENDIX

Molecular pathways of senescence regulate placental structure and function

Hilah Gal, Marina Lysenko, Sima Stroganov, Ezra Vadai, Sameh A.Youssef, Keren Tzadikevitch-Geffen, Ron Rotkopf, Tal Biron-Shental, Alain de Bruin, Michal Neeman and Valery Krizhanovsky

Table of contents

Appendix Figure S1. Contrast agent biotin-BSA-GdDTPA is localized in the labyrinth of the murine placenta following dynamic contrast-enhanced (DCE)-MRI.

Appendix Figure S2. Placentas with attenuated senescence programs exhibit an altered signal intensity (SI) dynamics, studied by in-utero DCE-MRI.

Appendix Figure S3. Murine placentas of Cdkn2a$^{-/-}$;p53$^{-/-}$ exhibit disruption of vasculature in the labyrinth.

Appendix Figure S4. Murine placentas with attenuated senescence programs exhibit morphological anomalies in the trophospongium.

Appendix Figure S5. The syncytiotrophoblast-containing labyrinth of the murine WT placenta expresses markers of cellular senescence.

Appendix Figure S6. Murine placentas express markers of cellular senescence p53 and ARF in the labyrinth syncytiotrophoblast.

Appendix Figure S7. Murine cytotrophoblasts exhibit increased proliferation in the labyrinth of Cdkn2a$^{-/-}$;p53$^{-/-}$ placenta.

Appendix Figure S8. SA-β-gal activity in the murine placental labyrinth of *Cdkn1a*$^{-/-}$ and *p53*$^{-/-}$ mice.

Appendix Figure S9. Human primary trophoblasts differentiate in culture and express markers of syncytiotrophoblast.

Appendix Figure S10. The βHCG hormone is exclusively expressed in the syncytiotrophoblast of the human placenta.

Appendix Figure S11. Expression matrix of 204 modulated genes after superparamagnetic clustering (SPC) of differentially expressed genes in human primary trophoblast cultures.

Appendix Table S1. Clinical characteristics of IUGR patients.

Appendix Table S2. List of upregulated genes (Fold change ≥1.8, p<0.05).

Appendix Table S3. List of downregulated genes (fold change ≤ -1.8, p<0.05).

Appendix Table S4. GSEA groups of genes.

Appendix Table S5 List of antibodies.

Appendix Supplementary Methods

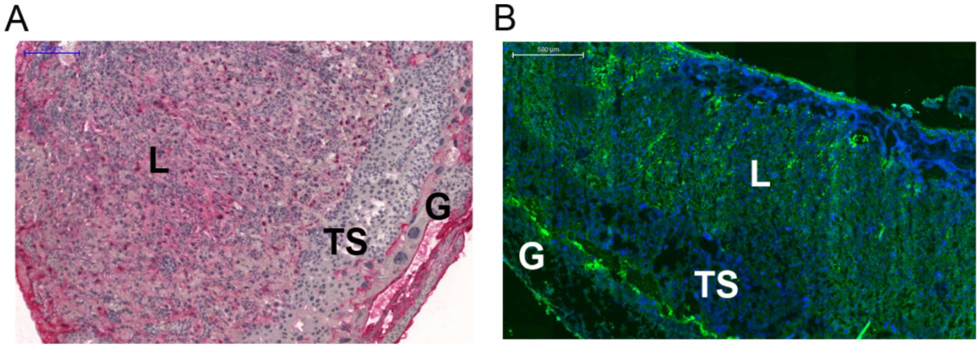

Appendix Figure S1. Contrast agent biotin-BSA-GdDTPA is localized in the labyrinth of the murine placenta following dynamic contrast-enhanced (DCE)-MRI.

(A, B) Placentas were retrieved from pregnant C57BL/6 WT mice on day E14.5, 60 min after injection of biotin-BSA-GdDTPA (10 mg/kg) via the tail vein. (A) Contrast agent staining with avidin-alkaline phosphatase: red. Scale bar, 200 µm. (B) Contrast agent staining with avidin-FITC: blue, nuclear staining with DAPI; green, avidin-FITC. L, labyrinth, TS, trophospongium, G, giant cells. Scale bar, 500 µm.

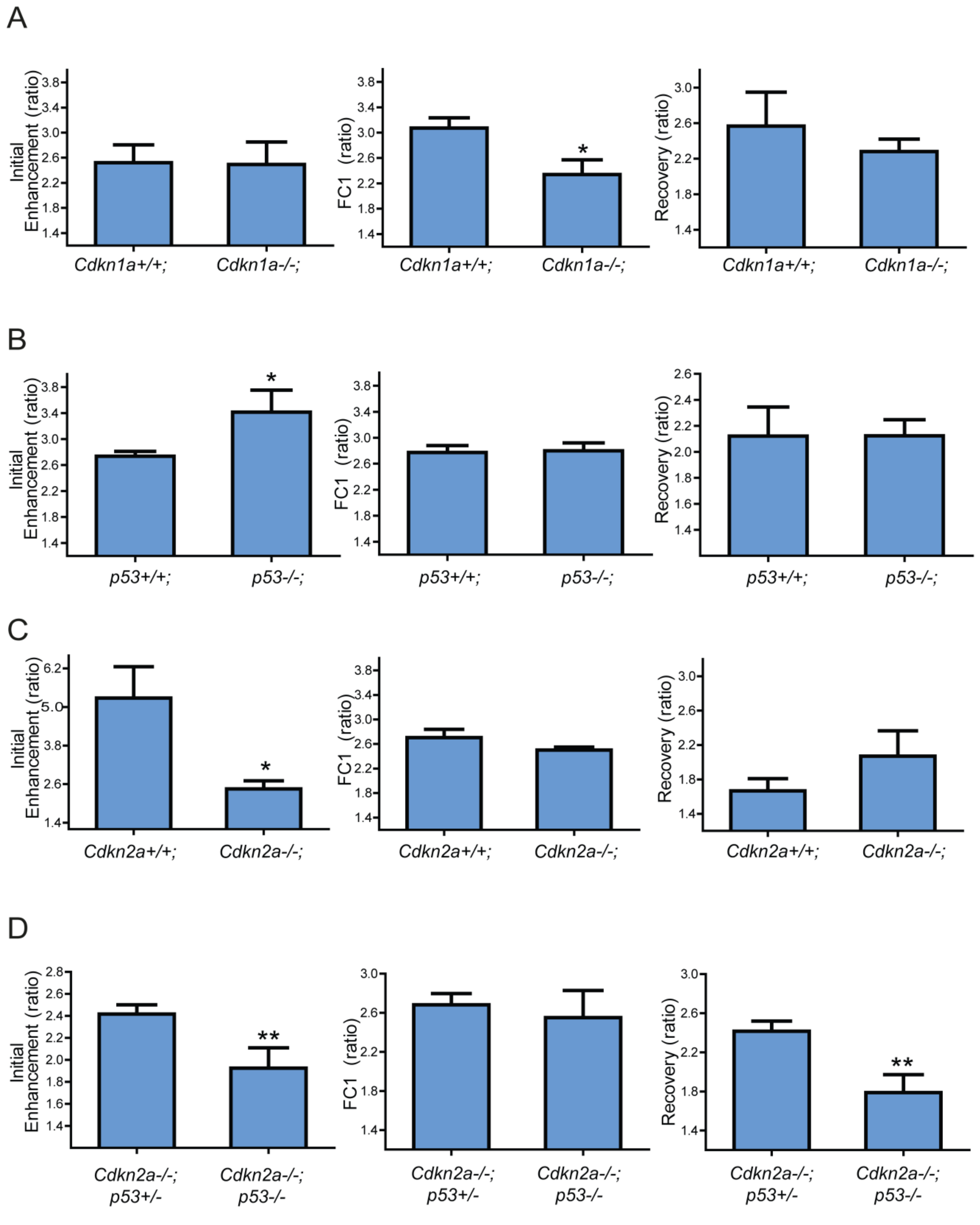

Appendix Figure S2. Placentas with attenuated senescence programs exhibit altered signal intensity (SI) dynamics, studied by in-utero DCE-MRI.. DCE-MRI was performed in pregnant mice of *Cdkn1a, p53, Cdkn2a* and *Cdkn2a;p53* genotypes on day E14.5. Mice were injected i.v. with an albumin-labeled contrast agent (biotin-BSA-GdDTPA; 10 mg/mouse) and placental enhancement was monitored at 9.4T MRI. Quantification of the SI parameters

Initial Enhancement[#], FC1[##] and Recovery[###] in (A) *Cdkn1a$^{-/-}$, (B) p53$^{-/-}$, (C) Cdkn2a$^{-/-}$,* and (D) *Cdkn2a$^{-/-}$;p53$^{-/-}$* placentas and in their respective WT or heterozygous littermates. MRI experiments were repeated at least three times for each murine genotype (*Cdkn1a*, n = 3), (*p53,* n = 4), (*Cdkn2a*, n = 3) and (*Cdkn2a;p53*], n = 4). Values are means + SEM; (*) $p < 0.05$; (**) $p < 0.01$. [#]Initial enhancement: ratio of the first SI maximum to the initial SI point. [##]FC1: ratio of the SI minimum point between the two maximum points to the first SI maximum. [###]Recovery: ratio of the second SI maximum to the SI minimum between the two maximum points.

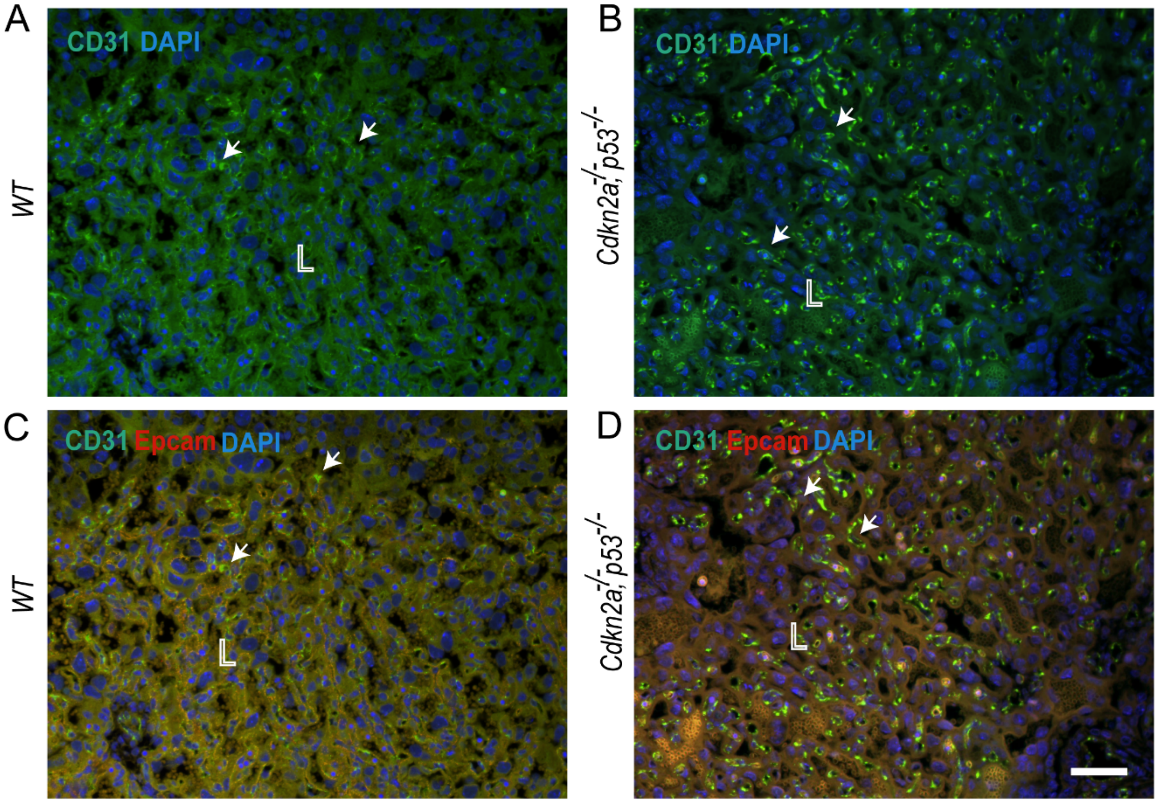

Appendix Figure S3. Murine placentas of Cdkn2a$^{-/-}$;p53$^{-/-}$ exhibit disruption of vasculature in the labyrinth. Immunofluorescence staining of endothelial cells (CD31) in the labyrinth zone of the murine *Cdkn2a$^{-/-}$;p53$^{-/-}$* compared to wild type (WT) on E14.5. (A,B) The *Cdkn2a$^{-/-}$;p53$^{-/-}$* placenta have smaller blood vessel lumina in the labyrinth and appear more compressed compared to wild-type. In addition, *Cdkn2a$^{-/-}$;p53$^{-/-}$* blood vessels are not equally distributed compared to the wildtype vessel in the labyrinth. (C,D) *Cdkn2a$^{-/-}$;p53$^{-/-}$* labyrinth exhibit more intense Epcam staining compared to WT, most likely illustrating the hyperplasia of the labyrinth trophoblasts. Green label: Endothelial marker CD31, Red label: Epcam, Blue label: DAPI nuclear stain. Arrowheads indicate positively stained CD31 cells. Scale bar, 100 µm.

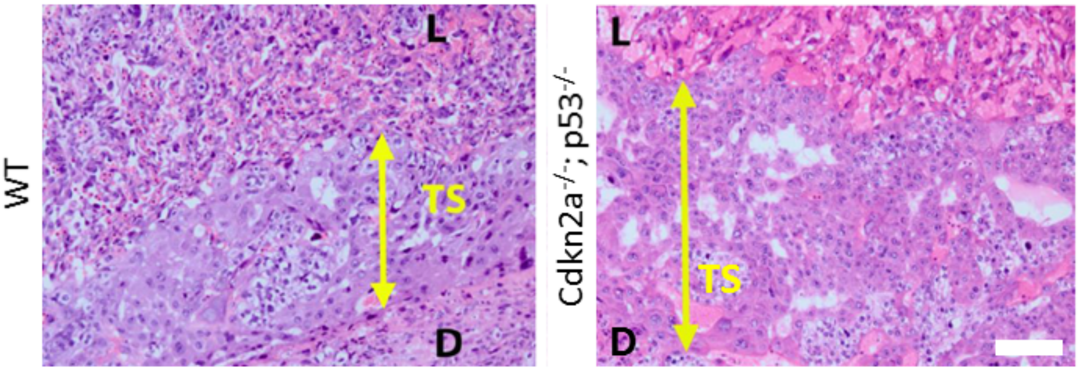

Appendix Figure S4. Murine placentas with attenuated senescence programs exhibit morphological anomalies in the trophospongium. Histological evaluation of H&E-stained sections of murine WT (left) and *Cdkn2a*$^{-/-}$ *;p53*$^{-/-}$ *(*right*)* on E14.5. reveals differences in the thinkness of the trophospongium zone (yellow double-headed arrow). The TS.zone in the *Cdkn2a*$^{-/-}$*;p53*$^{-/-}$ placenta is significantly thicker and appears more cellular. L= Labyrinth, TS= trophospongium, D=decidua. Scale bars, 100 μm.

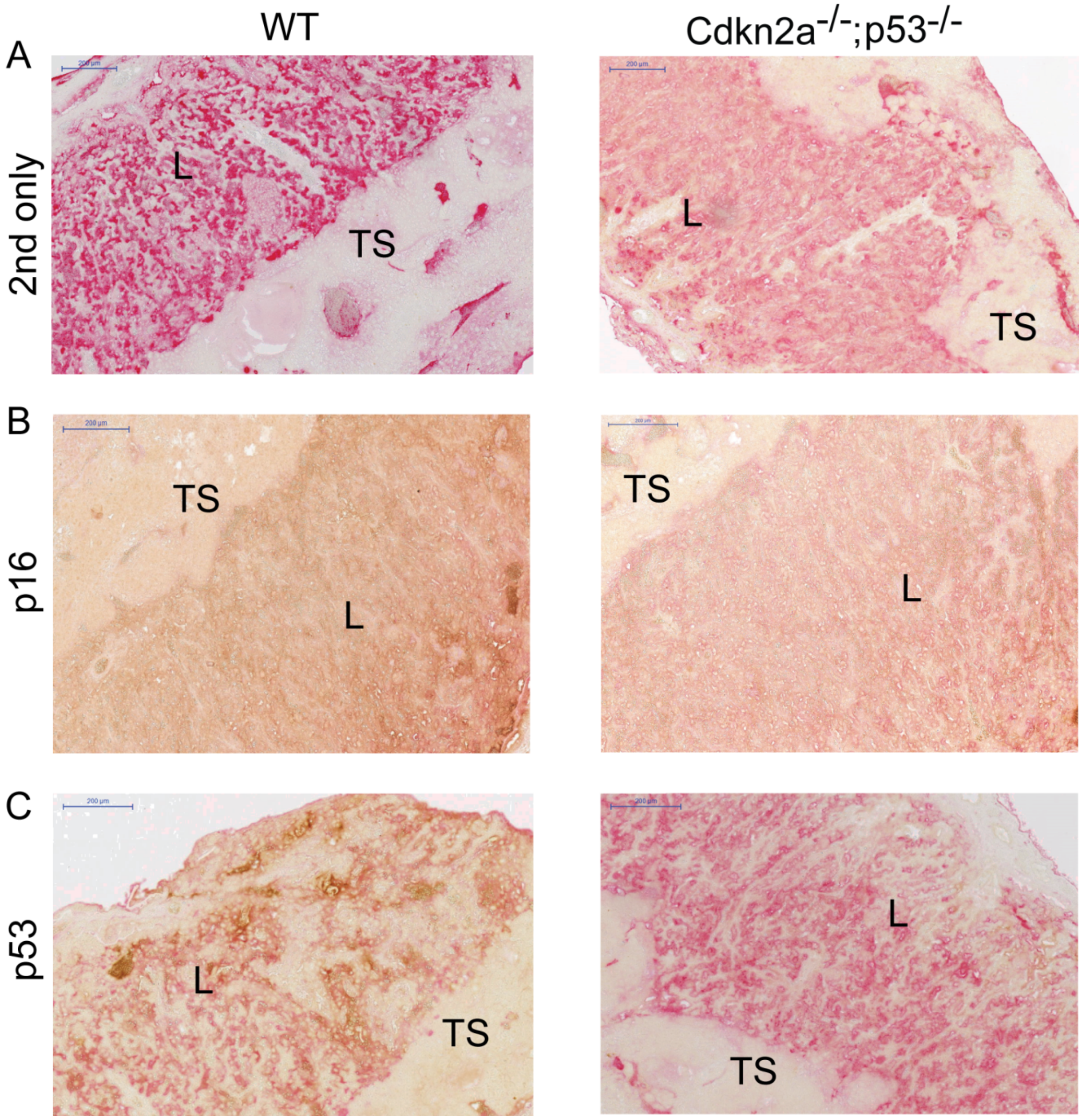

Appendix Figure S5. The syncytiotrophoblast-containing labyrinth of the murine WT placenta expresses markers of cellular senescence. Placental sections were derived from pregnant WT and *Cdkn2a$^{-/-}$;p53$^{-/-}$* mice (day E14.5) after injection of biotin-BSA-GdDTPA (10 mg/kg) and MRI scanning. Sections were stained with avidin-alkaline phosphatase for detection of biotin-BSA-Gd-DTPA staining (red) (A) and with antibodies for staining of the senescence markers p16 (B) and p53 (C) (both brown). Scale bars, 200 μm, L = labyrinth, TS = trophospongium.

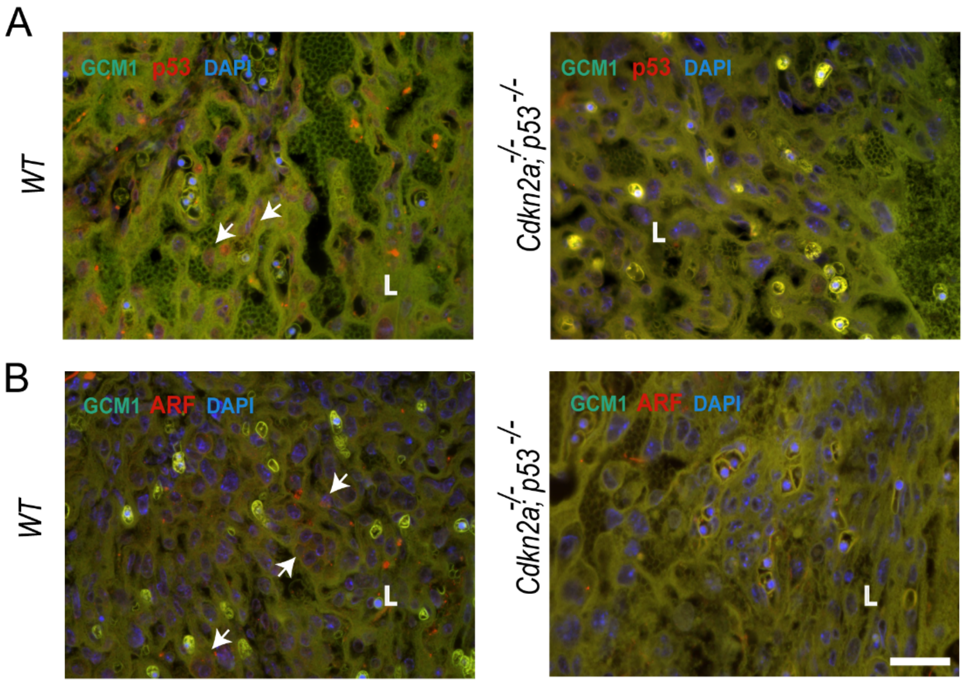

Appendix Figure S6. Murine placentas express markers of cellular senescence p53 and ARF in the labyrinth syncytiotrophoblast. Immunofluorescence staining of the labyrinth zone of the murine *Cdkn2a*$^{-/-}$*;p53*$^{-/-}$ compared to wild type (WT) on E14.5. (A) p53 and (B) ARF are expressed in the labyrinth syncytiotrophoblast of WT, but not *Cdkn2a*$^{-/-}$*;p53*$^{-/-}$ placenta. Green label: syncytiotrophoblast marker GCM1, Red label: p53 or ARF. Blue label: DAPI nuclear stain. Arrowheads indicate positively stained cells ((A,C) GCM1^{+}/p53^{+}, (B) GCM1^{+}/ARF^{+}. Scale bar, 50 μm.

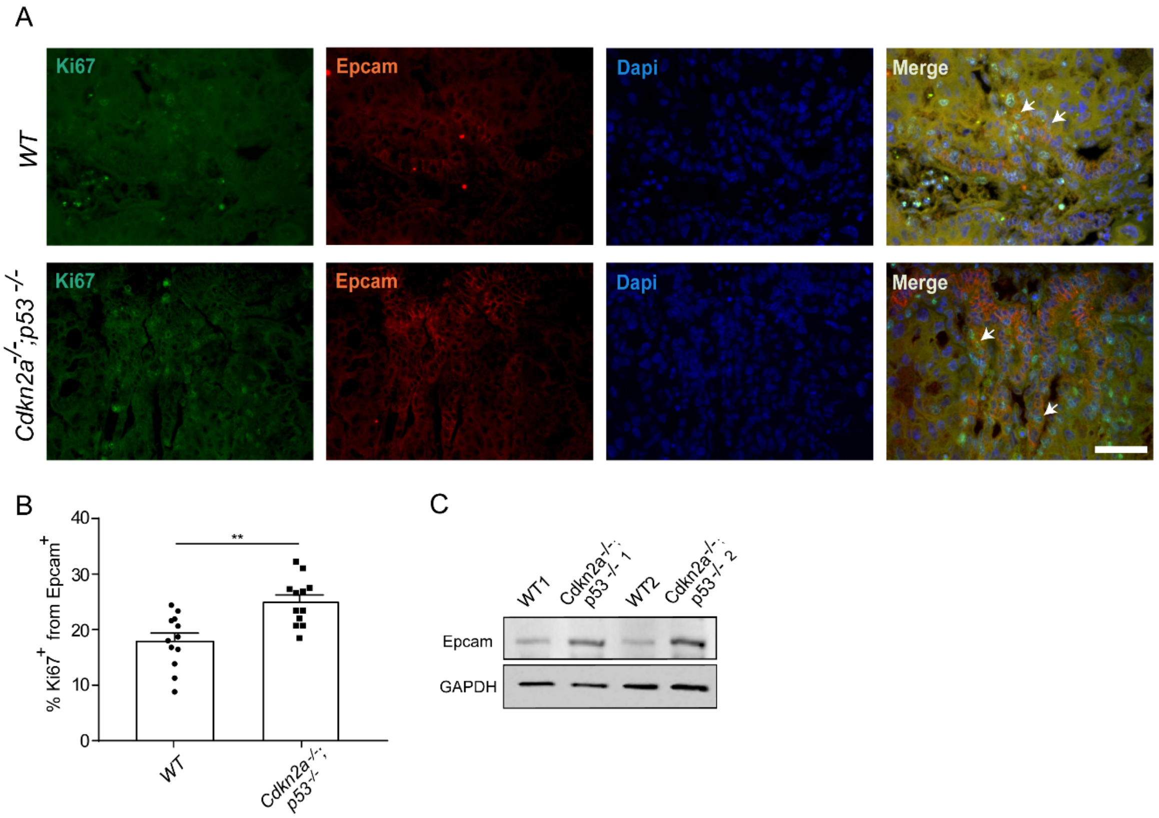

Appendix Figure S7 Murine cytotrophoblasts exhibit increased proliferation in the labyrinth of Cdkn2a$^{-/-}$;p53$^{-/-}$ placenta. A) Representative images of Immunofluorescence co-staining for Ki67 proliferation marker and the labyrinth progenitor trophoblast marker, Epcam, in the murine *Cdkn2a$^{-/-}$;p53$^{-/-}$* labyrinth compared to WT, on E14.5. Green label: Ki67, Red label: Epcam. Blue label: DAPI nuclear stain. Arrowheads indicate positively stained Epcam+Ki67+ cells. Scale bar, 50 µm. B) Quantification based on ki67 and Epcam co-staining in the labyrinth (derived from n=12 fields of view from three placentas of each genotype) shows enhanced KI67 expression in Epcam+ population of 25.0%±1.22 versus 17.9%±1.39 (for *Cdkn2a$^{-/-}$;p53$^{-/-}$* and WT respectively). Values are means +SEM. **P < 0.01 by a two-tailed unpaired Student's t-test. C) Immunoblot analysis exhibits elevation in protein content of Epcam in *Cdkn2a$^{-/-}$;p53$^{-/-}$* compared to WT placenta. Each lane represents one independent WT or *Cdkn2a$^{-/-}$;p53$^{-/-}$* placenta.

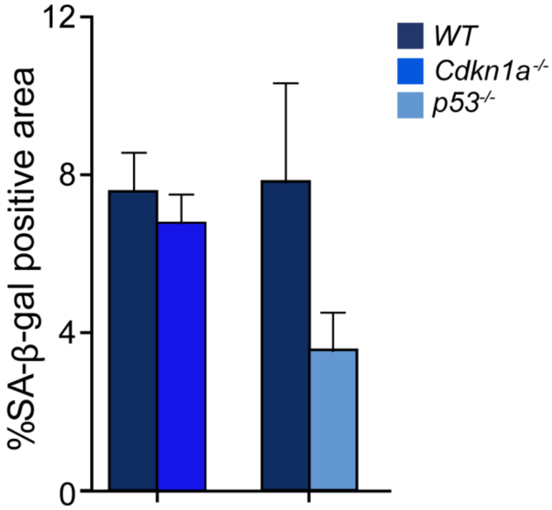

Appendix Figure S8. SA-β-gal activity in the murine placental labyrinth of *Cdkn1a*$^{-/-}$ and *p53*$^{-/-}$ mice.

Quantification of SA-β-gal activity in the placental labyrinth of *Cdkn1a*$^{-/-}$ and *p53*$^{-/-}$ mice and in WT mice. Percentages of SA-β-gal positive area are shown. Values are means + SEM of three experiments.

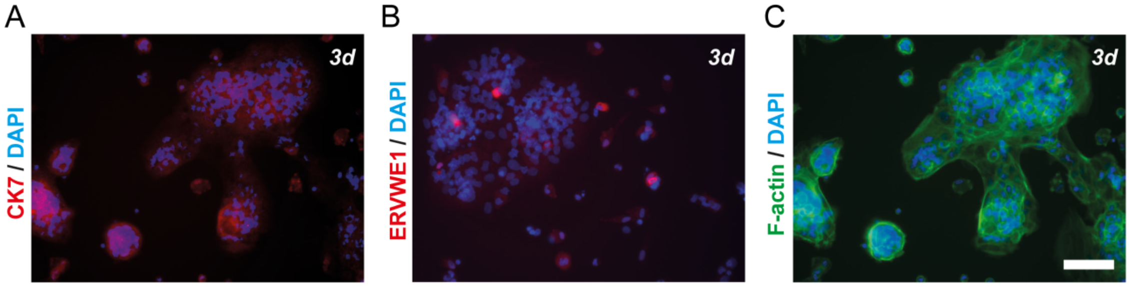

Appendix Figure S9. Human primary trophoblasts differentiate in culture and express markers of syncytiotrophoblast.

Human term placentas were dissected and cytotrophoblast cells were extracted and seeded. (A, B) On day 3 after seeding, the identity of the seeded population was determined by staining for the trophoblast marker cytokeratin7 (CK7, red) (A) and the ERVWE1 fusogene (ERVWE1, red) (B). (C) On day 3 after seeding, cell fusion was monitored by staining for phalloidin (F-actin; green) and DAPI (blue). Scale bar, 50 μM.

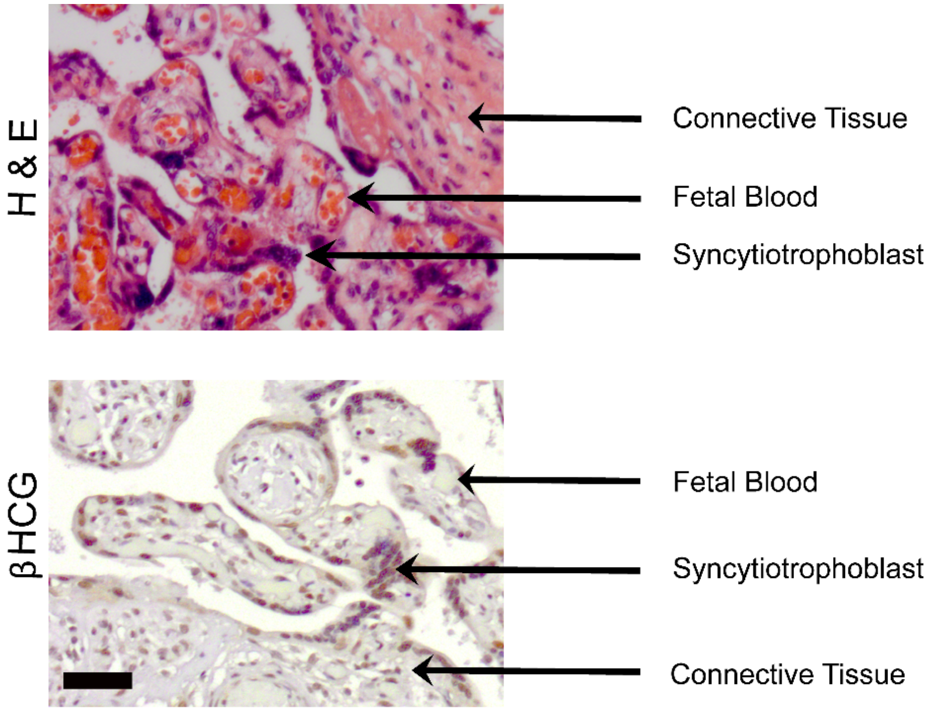

Appendix Figure S10. The βHCG hormone is exclusively expressed in the syncytiotrophoblast of the human placenta. Histological examination of human placenta morphology Sections of human post-partum third trimester placenta were evaluated by A) H&E staining, demonstrating the general structure of the placenta and by (B) Immunohistochemistry for the expression of the syncytiotrophoblast marker βHCG marker. Scale bar, 50 μm.

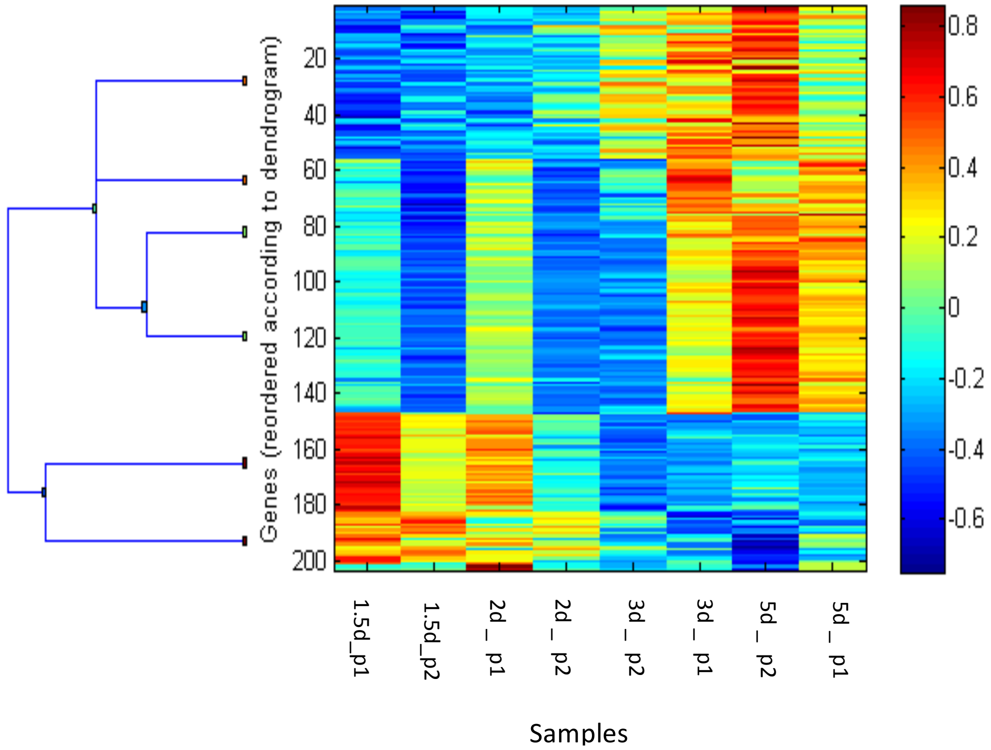

Appendix Figure S11. Expression matrix of 204 modulated genes after superparamagnetic clustering (SPC) of differentially expressed genes in human primary trophoblast cultures.

The expression data represent trophoblast cultures, derived independently from two human term placentas (labeled p1 and p2). Rows represent genes; columns represent samples. Blue denotes low expression; red denotes high expression.

Appendix Table S1. Clinical characteristics of IUGR patients

Clinical characteristics of the patients from whom study placentas were obtained:

	IUGR (n=10)	Control (n=10)	P value
Average gestational week at the delivery	37.0±2.83	37.25±2.6	NS
Average birth weight in grams	1881±350	3205±657	P<0.005
% of patients with Oligohydramnion	40	0	P<0.005
% of patients with umbilical Doppler abnormalities	45	0	P<0.005
% of patients with non-reassuring fetal heart rate	45	15	P<0.005
% samples with histological characteristics of placental insufficiency	100	0	P<0.005

* Redline RW. Placental pathology: a systematic approach with clinical correlations. Placenta 2008;29:S86–91.
* Salafia CM, Charles AK, Maas EM. Placenta and fetal growth restriction. Clin Obstet Gynecol 2006;49(2):236–56.

IUGR was defined as fetal weight below the 10th percentile for the gestational age. We included only placental specimens from pregnancies complicated with IUGR attributed to placental insufficiency, after ruling out other reasons for IUGR such as intrauterine viral infections, chromosomal abnormalities congenital anomalies, poor pregnancy dating, or maternal smoking. Furthermore, in the IUGR group, we included placentas from pregnancies with clinical characteristics of placental insufficiency such as oligohydramnios, umbilical artery abnormalities and non-reassuring fetal heart rates. We also assessed the prevalence of histopathologic characteristics that are typical to placental insufficiency including chronic villitis, perivillous fibrin deposition, villous hypoplasia, and infarcts with fetal and maternal vascular obstruction*, all of which were significantly more abundant in the IUGR placentas included in the study.

We certified that the average gestational age at delivery was similar between the IUGR and the control groups. The control samples were taken from low risk uneventful pregnancies with a thorough follow-up during the pregnancy including normal anomaly scans. Al the fetuses were evaluated as healthy babies during the first neonatal medical evaluation.

Appendix Table S2. List of upregulated genes (Fold change ≥1.8, p<0.05)

#	Probeset ID	Entrez Gene	Gene Symbol	Gene Title	P-Value	Fold-Change (Upregulated ≥ 1.8)
1	11758967_s_at	7980	TFPI2	tissue factor pathway inhibitor 2	0.0141	8.87
2	11737291_a_at	27199	OXGR1	oxoglutarate (alpha-ketoglutarate) receptor 1	0.0033	5.87
3	11755438_x_at	5671	PSG3	pregnancy specific beta-1-glycoprotein 3	0.0037	5.65
4	11757022_x_at	7980	TFPI2	tissue factor pathway inhibitor 2	0.0048	4.86
5	11715138_s_at	1082	CGB	chorionic gonadotropin, beta polypeptide	0.0019	4.66
6	11721995_a_at	2615	LRRC32	leucine rich repeat containing 32	0.0043	4.44
7	11743044_a_at	216	ALDH1A1	aldehyde dehydrogenase 1 family, member A1	0.0325	4.39
8	11727938_s_at	7869	SEMA3B	semaphorin 3B	0.0029	4.12
9	11761888_x_at	5678	PSG9	pregnancy specific beta-1-glycoprotein 9	0.0013	3.99
10	11727075_at	191585	PLAC4	placenta specific 4	0.0270	3.87
11	11750279_a_at	6781	STC1	stanniocalcin 1	0.0001	3.86
12	11754601_s_at	10417	SPON2	spondin 2, extracellular matrix protein	0.0195	3.53
13	11724197_at	6781	STC1	stanniocalcin 1	0.0005	3.38
14	11715455_x_at	3814	KISS1	KiSS-1 metastasis-suppressor	0.0374	3.32
15	11724198_a_at	6781	STC1	stanniocalcin 1	0.0002	3.30
16	11715454_at	3814	KISS1	KiSS-1 metastasis-suppressor	0.0317	3.29
17	11744491_x_at	7869	SEMA3B	semaphorin 3B	0.0025	3.23
18	11726855_at	11096	ADAMTS5	ADAM metallopeptidase with thrombospondin type 1 motif 5	0.0488	3.23
19	11716605_at	8038	ADAM12	ADAM metallopeptidase domain 12	0.0092	3.21
20	11723245_a_at	6422	SFRP1	secreted frizzled-related protein 1	0.0009	3.13
21	11727836_a_at	8532	CPZ /// GPR78	carboxypeptidase Z /// G protein-coupled receptor 78	0.0063	3.10
22	11722379_at	2791	GNG11	guanine nucleotide binding protein (G protein), gamma 11	0.0048	3.09
23	11715949_s_at	115207	KCTD12	potassium channel tetramerization domain containing 12	0.0408	3.05
24	11756471_a_at	84879	MFSD2A	major facilitator superfamily domain containing 2A	0.0032	2.98
25	11743742_at	23250	ATP11A	ATPase, class VI, type 11A	0.0111	2.92
26	11718766_at	11098	PRSS23	protease, serine, 23	0.0297	2.90
27	11717986_a_at	341	APOC1	apolipoprotein C-I	0.0356	2.85
28	11752539_a_at	5669	PSG1	pregnancy specific beta-1-glycoprotein 1	0.0062	2.82
29	11746910_x_at	203100	HTRA4	HtrA serine peptidase 4	0.0027	2.77
30	11756157_x_at	5670	PSG2	pregnancy specific beta-1-glycoprotein 2	0.0031	2.66
31	11736579_a_at	2689	GH2	growth hormone 2	0.0105	2.64
32	11723097_a_at	79679	VTCN1	V-set domain containing T cell activation inhibitor 1	0.0398	2.62
33	11724668_at	3641	INSL4	insulin-like 4 (placenta)	0.0465	2.60
34	11743172_a_at	28231	SLCO4A1	solute carrier organic anion transporter family, 4A1	0.0428	2.58
35	11739851_a_at	203100	HTRA4	HtrA serine peptidase 4	0.0023	2.57
36	11758166_s_at	3730	ANOS1	anosmin 1	0.0241	2.56
37	11727947_at	1953	MEGF6	multiple EGF-like-domains 6	0.0179	2.51
38	11756149_x_at	5675	PSG6	pregnancy specific beta-1-glycoprotein 6	0.0271	2.47
39	11744584_a_at	81888	HYI	hydroxypyruvate isomerase (putative)	0.0023	2.45
40	11723829_s_at	55107	ANO1	anoctamin 1, calcium activated chloride channel	0.0029	2.45
41	11724392_at	57168	ASPHD2	aspartate beta-hydroxylase domain containing 2	0.0226	2.45
42	11739641_a_at	60312	AFAP1	actin filament associated protein 1	0.0018	2.37
43	11753610_a_at	79679	VTCN1	V-set domain containing T cell activation inhibitor 1	0.0085	2.36

44	11728991_a_at	57722	IGDCC4	immunoglobulin superfamily, DCC subclass, member 4	0.0273	2.36
45	11730353_a_at	56603	CYP26B1	cytochrome P450, family 26, subfamily B, polypeptide 1	0.0224	2.35
46	11748648_a_at	5801	PTPRR	protein tyrosine phosphatase, receptor type, R	0.0308	2.33
47	11726337_a_at	240	ALOX5	arachidonate 5-lipoxygenase	0.0207	2.33
48	11756509_a_at	25805	BAMBI	BMP and activin membrane-bound inhibitor	0.0012	2.32
49	11744842_at	58483	LINC00474	long intergenic non-protein coding RNA 474	0.0317	2.31
50	11732657_a_at	1592	CYP26A1	cytochrome P450, family 26, subfamily A, polypeptide 1	0.0149	2.28
51	11749869_x_at	79679	VTCN1	V-set domain containing T cell activation inhibitor 1	0.0297	2.26
52	11728992_s_at	57722	IGDCC4	immunoglobulin superfamily, DCC subclass, member 4	0.0156	2.25
53	11724566_a_at	83660	TLN2	talin 2	0.0039	2.25
54	11759497_at	50515	CHST11	carbohydrate (chondroitin 4) sulfotransferase 11	0.0444	2.23
55	11755772_a_at	2308	FOXO1	forkhead box O1	0.0035	2.21
56	11736030_a_at	9123	SLC16A3	solute carrier family 16, member 3	0.0045	2.20
57	11724249_a_at	64116	SLC39A8	solute carrier family 39 (zinc transporter), member 8	0.0107	2.20
58	11742948_at	4082	MARCKS	myristoylated alanine-rich protein kinase C substrate	0.0321	2.18
59	11720008_a_at	79689	STEAP4	STEAP family member 4	0.0042	2.17
60	11723111_a_at	84034	EMILIN2	elastin microfibril interfacer 2	0.0366	2.16
61	11716887_a_at	4072	EPCAM	epithelial cell adhesion molecule	0.0408	2.16
62	11746931_a_at	3624	INHBA	inhibin beta A	0.0131	2.16
63	11747671_x_at	1397	CRIP2	cysteine-rich protein 2	0.0361	2.15
64	11724006_a_at	953	ENTPD1	ectonucleoside triphosphate diphosphohydrolase 1	0.0307	2.14
65	11720007_a_at	79689	STEAP4	STEAP family member 4	0.0099	2.13
66	11716015_a_at	123920	CMTM3	CKLF-like MARVEL transmembrane domain containing 3	0.0067	2.13
67	11728085_s_at	658	BMPR1B	bone morphogenetic protein receptor type IB	0.0301	2.13
68	11718198_at	10186	LHFP	lipoma HMGIC fusion partner	0.0190	2.13
69	11750736_a_at	79689	STEAP4	STEAP family member 4	0.0098	2.12
70	11735090_s_at	9120	SLC16A6	solute carrier family 16, member 6	0.0128	2.11
71	11723576_s_at	116039	OSR2	odd-skipped related transciption factor 2	0.0021	2.11
72	11728434_a_at	2689	GH2	growth hormone 2	0.0028	2.10
73	11722743_a_at	7089	TLE2	transducin-like enhancer of split 2	0.0002	2.09
74	11742938_at	430	ASCL2	achaete-scute family bHLH transcription factor 2	0.0002	2.08
75	11731496_at	3624	INHBA	inhibin beta A	0.0341	2.07
76	11740208_x_at	151556	GPR155	G protein-coupled receptor 155	0.0070	2.06
77	11729227_a_at	2869	GRK5	G protein-coupled receptor kinase 5	0.0020	2.05
78	11749188_a_at	953	ENTPD1	ectonucleoside triphosphate diphosphohydrolase 1	0.0349	2.02
79	11723188_s_at	8728	ADAM19	ADAM metallopeptidase domain 19	0.0307	2.02
80	11741579_a_at	7780	SLC30A2	solute carrier family 30 (zinc transporter), member 2	0.0013	2.01
81	11723389_a_at	404550	C16orf74	chromosome 16 open reading frame 74	0.0012	1.99
82	11743108_a_at	58	ACTA1	actin, alpha 1, skeletal muscle	0.0452	1.99
83	11757654_x_at	7351	UCP2	uncoupling protein 2 (mitochondrial, proton carrier)	0.0250	1.99
84	11727929_a_at	2308	FOXO1	forkhead box O1	0.0063	1.99
85	11727622_a_at	5212	VIT	vitrin	0.0132	1.98
86	11715205_x_at	5672	PSG4	pregnancy specific beta-1-glycoprotein 4	0.0061	1.96
87	11733406_a_at	149461	CLDN19	claudin 19	0.0432	1.96
88	11734704_at	3910	LAMA4	laminin, alpha 4	0.0439	1.95
89	11724454_at	6583	SLC22A4	solute carrier family 22, member 4	0.0055	1.95
90	11744321_a_at	79816	TLE6	transducin-like enhancer of split 6	0.0154	1.94

91	11721124_s_at	4320	MMP11	matrix metallopeptidase 11	0.0012	1.94
92	11740475_x_at	50507	NOX4	NADPH oxidase 4	0.0156	1.93
93	11729610_a_at	9844	ELMO1	engulfment and cell motility 1	0.0299	1.92
94	11734720_a_at	8612	PLPP2	phospholipid phosphatase 2	0.0115	1.91
95	11746639_a_at	9770	RASSF2	Ras association (RalGDS/AF-6) domain family member 2	0.0374	1.89
96	11728531_a_at	79816	TLE6	transducin-like enhancer of split 6	0.0182	1.88
97	11738880_x_at	440533	PSG8	pregnancy specific beta-1-glycoprotein 8	0.0165	1.88
98	11756810_a_at	4237	MFAP2	microfibrillar associated protein 2	0.0120	1.87
99	11723836_a_at	124976	SPNS2	spinster homolog 2 (Drosophila)	0.0380	1.87
100	11718955_a_at	54751	FBLIM1	filamin binding LIM protein 1	0.0346	1.87
101	11718630_s_at	666	BOK	BCL2-related ovarian killer	0.0274	1.86
102	11729887_at	9200	HACD1	3-hydroxyacyl-CoA dehydratase 1	0.0140	1.86
103	11727221_at	64131	XYLT1	xylosyltransferase I	0.0065	1.85
104	11716771_s_at	150094	SIK1	serine/threonine-protein kinase SIK1	0.0177	1.85
105	11718513_x_at	81619	TSPAN14	tetraspanin 14	0.0021	1.84
106	11732221_at	1392	CRH	corticotropin releasing hormone	0.0400	1.83
107	11736461_at	5865	RAB3B	RAB3B, member RAS oncogene family	0.0033	1.83
108	11726278_at	8303	SNN	stannin	0.0443	1.82

Appendix Table S3. List of downregulated genes (fold change ≤ -1.8, p<0.05)

#	Probeset ID	Entrez Gene	Gene Symbol	Gene Title	P-Value	Fold-Change (down-regulated ≤ -1.8)
1	11758138_s_at	9656	MDC1	mediator of DNA-damage checkpoint 1	0.0461	-1.80
2	11754594_a_at	1836	SLC26A2	solute carrier family 26 (anion exchanger), member 2	0.0274	-1.80
3	11745948_a_at	1111	CHEK1	checkpoint kinase 1	0.0467	-1.81
4	11733745_a_at	2651	GCNT2	glucosaminyl (N-acetyl) transferase 2, I-branching enzyme	0.0014	-1.81
5	11736935_x_at	25833	POU2F3	POU class 2 homeobox 3	0.0321	-1.81
6	11716117_at	9601	PDIA4	protein disulfide isomerase family A, member 4	0.0366	-1.82
7	11748602_a_at	9532	BAG2	BCL2-associated athanogene 2	0.0195	-1.84
8	11722818_a_at	8836	GGH	gamma-glutamyl hydrolase	0.0060	-1.84
9	11751073_a_at	25833	POU2F3	POU class 2 homeobox 3	0.0364	-1.85
10	11729038_a_at	51473	DCDC2	doublecortin domain containing 2	0.0321	-1.85
11	11725788_a_at	55839	CENPN	centromere protein N	0.0377	-1.86
12	11720105_a_at	1836	SLC26A2	solute carrier family 26 (anion exchanger), member 2	0.0292	-1.87
13	11733874_a_at	10051	SMC4	structural maintenance of chromosomes 4	0.0179	-1.88
14	11720319_at	10615	SPAG5	sperm associated antigen 5	0.0022	-1.89
15	11743530_a_at	64105	CENPK	centromere protein K	0.0335	-1.89
16	11723950_a_at	55872	PBK	PDZ binding kinase	0.0370	-1.89
17	11732860_at	221718	LINC00518	long intergenic non-protein coding RNA 518	0.0279	-1.90
18	11756855_a_at	2651	GCNT2	glucosaminyl	0.0020	-1.91
19	11718127_at	7083	TK1	thymidine kinase 1, soluble	0.0290	-1.91
20	11715257_x_at	170626	XAGE3	X antigen family, member 3	0.0499	-1.91
21	11721145_s_at	4288	MKI67	marker of proliferation Ki-67	0.0209	-1.92
22	11715788_a_at	30061	SLC40A1	solute carrier family 40, member 1	0.0458	-1.92
23	11733875_a_at	10051	SMC4	structural maintenance of chromosomes 4	0.0344	-1.92
24	11722069_a_at	220134	SKA1	spindle and kinetochore associated complex subunit 1	0.0323	-1.92
25	11750441_a_at	4001	LMNB1	lamin B1	0.0454	-1.92
26	11727124_s_at	4036	LRP2	LDL receptor related protein 2	0.0083	-1.93
27	11758149_s_at	29127	RACGAP1	Rac GTPase activating protein 1	0.0254	-1.94
28	11751976_a_at	4036	LRP2	LDL receptor related protein 2	0.0002	-1.94
29	11734953_at	55796	MBNL3	muscleblind-like splicing regulator 3	0.0377	-1.94
30	11718943_a_at	6790	AURKA	aurora kinase A	0.0213	-1.95
31	11756910_x_at	2177	FANCD2	Fanconi anemia complementation group D2	0.0142	-1.96
32	11742813_at	57556	SEMA6A	semaphorin 6A	0.0203	-1.97
33	11726029_a_at	7348	UPK1B	uroplakin 1B	0.0463	-1.99
34	11720647_a_at	701	BUB1B	BUB1 mitotic checkpoint serine/threonine kinase B	0.0267	-2.00
35	11754606_a_at	7184	HSP90B1	heat shock protein 90kDa beta (Grp94), member 1	0.0037	-2.01
36	11730167_at	2520	GAST	gastrin	0.0041	-2.01
37	11742815_x_at	57556	SEMA6A	semaphorin 6A	0.0200	-2.01
38	11715256_at	170626	XAGE3	X antigen family, member 3	0.0378	-2.01
39	11715789_a_at	30061	SLC40A1	solute carrier family 40, member 1	0.0340	-2.03
40	11716033_at	6590	SLPI	secretory leukocyte peptidase inhibitor	0.0169	-2.04
41	11718128_x_at	7083	TK1	thymidine kinase 1, soluble	0.0251	-2.05
42	11728377_x_at	10874	NMU	neuromedin U	0.0036	-2.05
43	11745361_a_at	643911	CRNDE	colorectal neoplasia differentially expressed	0.0086	-2.11
44	11733915_a_at	1825	DSC3	desmocollin 3	0.0224	-2.13
45	11730385_at	64388	GREM2	gremlin 2, DAN family BMP antagonist	0.0314	-2.15
46	11723583_a_at	29127	RACGAP1	Rac GTPase activating protein 1	0.0092	-2.16
47	11720203_a_at	9212	AURKB	aurora kinase B	0.0116	-2.17
48	11724209_a_at	3833	KIFC1	kinesin family member C1	0.0143	-2.20
49	11722571_at	890	CCNA2	cyclin A2	0.0170	-2.21

50	11730264_a_at	81610	FAM83D	family with sequence similarity 83, member D	0.0068	-2.22
51	11743296_a_at	1063	CENPF	centromere protein F	0.0096	-2.30
52	11718213_a_at	11001	SLC27A2	solute carrier family 27 (fatty acid transporter), member 2	0.0435	-2.35
53	11721932_a_at	9493	KIF23	kinesin family member 23	0.0046	-2.39
54	11722253_a_at	4751	NEK2	NIMA-related kinase 2	0.0080	-2.39
55	11752432_s_at	250	ALPP	alkaline phosphatase, placental	0.0098	-2.43
56	11747720_a_at	332	BIRC5	baculoviral IAP repeat containing 5	0.0082	-2.43
57	11729129_a_at	51200	CPA4	carboxypeptidase A4	0.0051	-2.45
58	11718058_a_at	7298	TYMS	thymidylate synthetase	0.0388	-2.57
59	11751805_a_at	7298	TYMS	thymidylate synthetase	0.0421	-2.58
60	11750824_a_at	9055	PRC1	protein regulator of cytokinesis 1	0.0029	-2.59
61	11745868_a_at	11004	KIF2C	kinesin family member 2C	0.0242	-2.59
62	11737016_a_at	4660	PPP1R12B	protein phosphatase 1, regulatory subunit 12B	0.0265	-2.66
63	11744789_a_at	79682	CENPU	centromere protein U	0.0372	-2.70
64	11742832_a_at	259266	ASPM	abnormal spindle microtubule assembly	0.0071	-2.71
65	11743687_s_at	3148	HMGB2	high mobility group box 2	0.0089	-2.77
66	11759328_at	57082	CASC5	cancer susceptibility candidate 5	0.0029	-2.82
67	11745464_x_at	1033	CDKN3	cyclin-dependent kinase inhibitor 3	0.0397	-2.87
68	11727543_at	11339	OIP5	Opa interacting protein 5	0.0215	-2.90
69	11717521_x_at	9232	PTTG1	pituitary tumor-transforming 1	0.0107	-2.95
70	11716793_a_at	9133	CCNB2	cyclin B2	0.0060	-2.96
71	11750598_s_at	22974	TPX2	TPX2, microtubule-associated	0.0174	-3.02
72	11756918_a_at	9493	KIF23	kinesin family member 23	0.0021	-3.04
73	11727489_a_at	3832	KIF11	kinesin family member 11	0.0280	-3.04
74	11724464_a_at	54443	ANLN	anillin actin binding protein	0.0103	-3.06
75	11744793_x_at	9787	DLGAP5	discs, large (Drosophila) homolog-associated protein 5	0.0364	-3.09
76	11717163_s_at	991	CDC20	cell division cycle 20	0.0109	-3.15
77	11740442_s_at	9582	APOBEC3A	apolipoprotein B mRNA editing enzyme, 3A	0.0069	-3.21
78	11716358_s_at	9055	PRC1	protein regulator of cytokinesis 1	0.0026	-3.22
79	11753788_x_at	1033	CDKN3	cyclin-dependent kinase inhibitor 3	0.0144	-3.28
80	11723435_s_at	7272	TTK	TTK protein kinase	0.0142	-3.36
81	11725625_s_at	59272	ACE2	angiotensin I converting enzyme 2	0.0032	-3.40
82	11742735_a_at	51203	NUSAP1	nucleolar and spindle associated protein 1	0.0215	-3.57
83	11748713_a_at	259266	ASPM	abnormal spindle microtubule assembly	0.0044	-3.71
84	11733702_x_at	11065	UBE2C	ubiquitin-conjugating enzyme E2C	0.0060	-3.85
85	11726617_s_at	983	CDK1	cyclin-dependent kinase 1	0.0111	-3.89
86	11744274_at	10403	NDC80	NDC80 kinetochore complex component	0.0238	-3.97
87	11725883_at	405754	ERVFRD-1	endogenous retrovirus group FRD, member 1	0.0258	-4.02
88	11732902_x_at	9582	APOBEC3B	apolipoprotein B mRNA editing enzyme, 3B	0.0035	-4.04
89	11758509_s_at	2028	ENPEP	glutamyl aminopeptidase (aminopeptidase A)	0.0004	-4.23
90	11732901_a_at	9582	APOBEC3A_B	apolipoprotein B mRNA editing enzyme, 3A	0.0034	-4.25
91	11723010_a_at	10112	KIF20A	kinesin family member 20A	0.0096	-4.30
92	11733884_a_at	55635	DEPDC1	DEP domain containing 1	0.0055	-4.30
93	11723565_a_at	1058	CENPA	centromere protein A	0.0074	-4.39
94	11758089_s_at	3161	HMMR	hyaluronan-mediated motility receptor (RHAMM)	0.0030	-4.52
95	11763384_a_at	2201	FBN2	fibrillin 2	0.0001	-4.72
96	11720970_at	7153	TOP2A	topoisomerase (DNA) II alpha	0.0011	-6.91

Appendix Table S4. GSEA lists of genes.

GSEA Group name	Nominal p-Value	Link to group
Activation of MAPK	P=0.004	http://software.broadinstitute.org/gsea/msigdb/cards/GO_ACTIVATION_OF_MAPK_ACTIVITY.html
Jak-Stat Cascade	P=0.016	http://software.broadinstitute.org/gsea/msigdb/cards/JAK_STAT_CASCADE.html
Immune response	P<0.001	http://software.broadinstitute.org/gsea/msigdb/cards/GO_IMMUNE_RESPONSE.html
Cytokine Activity	P<0.001	http://software.broadinstitute.org/gsea/msigdb/cards/CYTOKINE_ACTIVITY.html
Cell cycle process	P<0.001	http://software.broadinstitute.org/gsea/msigdb/cards/CELL_CYCLE_PROCESS.html
Mitosis	P<0.001	http://software.broadinstitute.org/gsea/msigdb/cards/MITOSIS.html
Aging	P<0.001	http://software.broadinstitute.org/gsea/msigdb/cards/DEMAGALHAES_AGING_UP.html
MMP activity	P=0.003	http://software.broadinstitute.org/gsea/msigdb/cards/METALLOPEPTIDASE_ACTIVITY.html
NFkB motif	P<0.001	http://software.broadinstitute.org/gsea/msigdb/cards/GGGNNTTTCC_NFKB_Q6_01.html
E2F targets	P<0.001	http://software.broadinstitute.org/gsea/msigdb/cards/HALLMARK_E2F_TARGETS.html
Pregnancy	P=0.022	http://software.broadinstitute.org/gsea/msigdb/cards/GO_MATERNAL_PROCESS_INVOLVED_IN_FEMALE_PREGNANCY.html
DNA replication	P<0.001	http://software.broadinstitute.org/gsea/msigdb/cards/DNA_REPLICATION.html

Appendix Table S5. List of Antibodies.

Antibody	Catalog #	Company	Purpose	IHC dilution
CDKN2B / p15INK4b	C0287	Assay Biotech	IHC	1:100
CDKN2A/p16INK4a	ab-108349	Abcam	IHC/WB	1:200/1:1000
P21 Waf/Cip (12D1)	C-2947	Cell signaling Technology	IHC	1:250
P21	PMG556431	BD Pharmingen	IHC/WB	1:50/1:1000
P53 (FL393)	sc-6243	Santa Cruz Biotechnology	IHC/WB	1:100/1:1000
EpCAM (C10)	sc-25308	Santa Cruz Biotechnology	IHC	1:100
EpCAM	Ab-71916	Abcam	IHC/WB	1:200/1:1000
CD31	Ab-124432	Abcam	IHC	1:500
Ki67	Ab-16667	Abcam	IHC	1:250
hCG beta	ab9582	Abcam	IHC	1:200
Syncitin (H-280)	sc-50369	Santa Cruz Biotechnology	IHC	1:100
CDKN2a/p19ARF	ab-80	Abcam	IHC	1:200
CDKN2A/p16INK4a	ab-54210	Abcam	IHC	1:500
P53	P53-CM5P-L	Leica	IHC	1:300
Epcam (G8.8)	sc- 53532	Santa Cruz Biotechnology	IHC	1:250
GCM1 (R06)	sc-101173	Santa Cruz Biotechnology	IHC	1:100
Mek 1/2	CST-9122	Cell signaling Technology	WB	1:1000
Phospho-Mek 1/2 (Ser217/221)	CST-9121	Cell signaling Technology	WB	1:1000
NFκB p65 (F-6)	sc-8008	Santa Cruz Biotechnology	WB	1:1000
Phospho-NFκB p65 (Ser536)	CST-3033	Cell signaling Technology	WB	1:1000
Stat3	CST-9139	Cell signaling Technology	WB	1:1000
Phospho-Stat3 (Tyr705)	CST-9145	Cell signaling Technology	WB	1:1000
Phospho-Smad3 (Ser423/425) (C25A9)	CST-9520	Cell signaling Technology	WB	1:1000
Smad3 (C67H9)	CST-9523	Cell signaling Technology	WB	1:1000
DCR2	adi-aap-371-e	Enzo	WB	1:1000
β-actin	A-5346	Sigma	WB	1:5000
GAPDH	MAB374	Merck	WB	1:5000

Appendix Supplementary Methods

Microarray analysis

Data analysis and preprocessing were carried out on human Affymetrix GeneChip PrimeView expression data (see Experimental Procedures). Partek Genomics Suite 6.6 (http://www.partek.com/) was used to identify the modulated genes in human primary trophoblast cultures on days 1.5, 2, 3, and 5 after seeding. Genes that were expressed below background levels (intensity of 5.4) in all examined conditions were excluded from further analyses. mRNA profiling identified 204 genes whose mean intensities in both placental cultures were significantly up- or downregulated (< / > 1.8-fold) on days 3 and 5 of culture, relative to their mean intensities on days 1.5 and 2. Differently expressed genes that passed the statistical one-way analysis of variance (ANOVA) with $P<0.05$ were selected. The modulated genes and samples were log2-transformed, normalized, and clustered using the superparamagnetic clustering (SPC) algorithm (Blatt, Wiseman et al., 1996).

Appendix Supplemental Reference

Blatt M, Wiseman S, Domany E (1996) Superparamagnetic clustering of data. Phys Rev Lett 76: 3251-3254
