# Supplementary material for: Molecular pathways of senescence regulate placental structure and function
Source: EMBO J. 2019 Aug 19;38(18):e100849. doi: 10.15252/embj.2018100849 (PMC6745498; doi:10.15252/embj.2018100849)
Supplement: Supplementary file 2 — Movie EV1 [file EMBJ-38-e100849-s002.zip › Movie_EV1_legend.docx]

Movie EV1**.** In-utero DCE-MRI of a WT mouse on day E14.5 of pregnancy, acquired during 60 min after administration of a contrast agent (biotin-BSA-GdDTPA).
